# Isolation of Microsatellite Markers in the *Calliptamus* Genus (Orthoptera, Acrididae)

**DOI:** 10.1673/031.010.13301

**Published:** 2010-08-13

**Authors:** E. Blanchet, C. Pages, L. Blondin, C. Billot, R. Rivallan, JM. Vassal, M. Lecoq, AM. Risterucci

**Affiliations:** ^1^ CIRAD, UPR Acridologie, Montpellier, F-34398 France; ^2^ CIRAD, UMR Development and Adaptation of Plants, Montpellier, F-34398 France

**Keywords:** Orthoptera, Calliptaminae, polymorphic

## Abstract

The *Calliptamus* genus (Orthoptera: Acrididae) includes locust and grasshopper species, some of which have a high economic impact. Using an enriched methodology, 10 microsatellite markers have been developed from two species, *Calliptamus italicus* and *Calliptamus barbarus.* These polymorphic markers were tested on different populations of three *Calliptamus* species: *C. italicus, C. barbarus, C. wattenwylianus.* Two markers were amplified on the three species, as well as four on *C. barbarus* and two on *C. italicus.* In each species, 9 to 23 alleles per locus were observed. These molecular markers might prove to be a new and interesting tool for *Calliptamus* population genetics and dispersion studies.

## Introduction

The genus *Calliptamus* (Orthoptera: Acrididae) includes locusts and grasshoppers, mainly found within the Mediterranean Basin to the southern part of Siberia. Some species of this genus are recognized as pests such as *C. italicus* (Linné, 1758) (pest occasionally of substantial importance), *C. barbarus* (Costa, 1836) (occasionally of localised importance), and *C. wattenwylianus* Pantel, 1896 (minor importance) (COPR 1982). Many studies have been conducted on their taxonomy and biology (Jago 1963; COPR 1982). However, despite their economic importance and the existence of migrations that could have an impact in outbreaks as is the case for other locusts (COPR 1982), there is a lack of knowledge on their dispersion capabilities. The objective of this work was to develop new molecular markers to enable genetic population studies and improve comprehension of dispersal ability of these pests and consequently of gene flow between different populations. Here is presented the development of first polymorphic microsatellites obtained from two DNA libraries (*C. italicus* and *C. barbarus*) which was tested on populations of three species of the *Calliptamus* genus: *C. italicus, C. barbarus,* and *C. wattenwylianus.*

## Materials and Methods

Microsatellite loci were isolated for *C. barbarus* and *C. italicus* from a genomic library enriched for di-nucleotide GA/CT and GT/CA following the protocol of Billote et al. (1999). Genomic DNA was extracted from the hind femur of one male of each species conserved in alcohol from the region of Languedoc (France) using a MATAB/PEG protocol (Risterucci et al. 2000).

Ten micrograms of extracted DNA were digested with Rsa I. Restriction fragments were ligated with adaptors (RSA21 and RSA25) and amplified. Microsatellite sequences were selected using biotin-labelled microsatellite oligoprobe and streptavidincoated magnetic beads. The selected fragments were amplified using RSA 21 primer, and resulting amplification products were cloned into pGEM-T vector (Promega, www.promega.com) and transformed into *Epicurian coli* XL1-Blue MRF supercompetent cells (Stratagene, www.genomics.agilent.com). Next, 192 recombinant colonies were amplified for each species, for 35 cycles using RSA 21 primer. PCR products were transferred onto nylon membranes (Hybond N+, Amersham, www.gelifesciences.com), and screening of clone containing microsatellites was made by hybrization using 5′ end ^32^P radiolabelled synthetic GA_15_ and GT_15_ microsatellite probes. Finally, 48 clones for each species showed a clear hybridization signal revealing a microsatellite and were chosen for sequencing.

Two different tools: SSR analysis (Dereeper et al. 2007) and Microfamily (Meglecz 2007) were used to observe flanking region similarity and length of microsatellite. Sequences that were similar or too short were eliminated. Then, using Primer 3 (Rozen and Skaletsky 1998), 18 (37.8%) primer pairs were designed for *C. barbarus,* and 15 (31%) for *C. italicus.*

Because an automated infrared fluorescence technology (4300; LI-COR Biosciences, www.licor.com) was used to detect each PCR product sample, an M13 (5′CACGACGT TGTAAAACGAC-3′) tailed primer (either IR700 or IR800 5′end labelled) was needed for PCR conditions.

PCR amplifications were run with 10 µl as the final volume, containing 4 ng of DNA, PCR buffer, 0.2 m*M* dNTPs, 1.5 m*M* MgCl_2_, 0.1 U Taq DNA polymerase, 0.2 µ*M* of primer, and 0.6 µ*M*of M13 tailed primer. PCR reactions were performed on the thermocycler (TC-412; Techne) following these parameters: denaturation at 94° C for 4 min; then 10 cycles with denaturation for 45 sec at 94° C and touch down from 60° C to 55° C or 63° C to 59° C in 1 min; and elongation time for 1 min followed by 35 cycles with denaturation for 45 sec at 94° C, hybridization for 1 min 15 sec at 55° or 59° C, and elongation at 72° C for 1 min; and the final step of elongation was operated for 4 min at 72° C.

Thirty males in total, from different localities for each species, were tested, in order to evaluate polymorphism and amplification with designed primer pairs (18 primer pairs from *C. barbarus* and 15 from *C. italicus*).

**Table 1.**
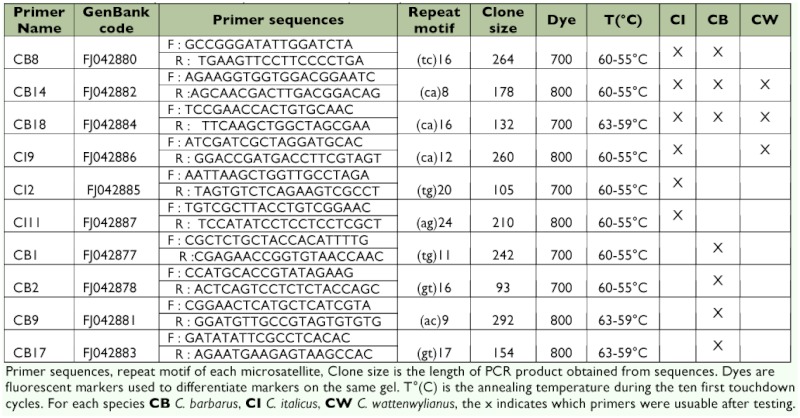
Characters of primers developed for three *Calliptamus* species.

Only males were used as females could not be morphologically determined at the species level. Insects were collected from two locations, Aumelas and Hortus, two karstic areas, 40 km apart, in Southern France. In each of these sites, 15 adults of *C. wattenwylianus,* 13 of C. *barbarus,* and 13 of *C. italicus* were collected. Five *C. barbarus* adults and three *C. italicus* were also collected in the Larzac plateau, a third location 40 km away from the two others. A last sample of two *C. italicus* adults was also available from Chizé, a location 400 km away.

## Results and Discussion

Finally, only seven primers from *C. barbarus* and three from *C. italicus* were kept, for their polymorphism and good amplification signal on different populations in each species. Two markers cross-amplified in the three species. One other cross-amplified in *C. italicus* and *C. barbarus,* and another on *C. italicus* and *C. wattenwylianus.* Two markers were species-specific for *C. italicus,* and the last four were species-specific for *C. barbarus.* Three markers were usual for *C. wattenwylianus;* seven for *C. barbarus,* and six for *C. italicus* (Table 1).

**Table 2.**
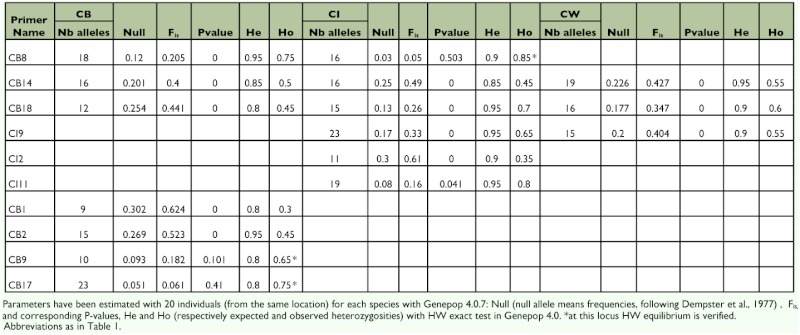
Statistical characters for each primer for each species.

Statistical analyses were conducted on 20 individuals from the same location for each species.

Linkage disequilibrium, allelic distribution, and heterozygosities were estimated with Genepop version 4. 0. 7 (Rousset 2008). No linkage disequilibrium between each pair of loci was found for any species. A high level of polymorphism was observed for common and specific markers. The number of alleles ranged for common markers from 12 to 23 per locus and from 9 to 23 per locus for specific markers (Table 2). The observed heterozygosity (Ho) varied by 0.3 to 0.85 and expected heterozygosity ranged from 0.8 to 0.95. Large differences between observed (Ho) and expected (He) heterozygosity (Table 2) could be explained by the presence of null alleles.

Analyses performed on Microchecker (van Oosterhout et al. 2006) showed that deficit in heterozygote was due to null alleles and not of genotyping errors. Null allele frequencies (Table 2) have been evaluated with Genepop version 4.0.7 (Rousset 2008), and their frequencies ranged from 0.05 to 0.4. In addition, certain insects frequently showed a high prevalence of null alleles, particularly Lepidoptera (Meglecz et al. 2004) and Orthoptera (Zhang et al. 2003; Chapuis et al. 2005; Yassin et al. 2006; Hamill et al. 2006; Chapuis et al. 2008a, 2008b).

It is advisable to redesign primer pairs or to develop other enriched bank to increase the number of markers. Although null alleles have an incidence on statistics and methods used in microsatellite analyses (Chapuis et al. 2008a), new tools such as those developed by Chapuis and Estoup (2007) should enable the use of these markers in population genetic studies on *Calliptamus.*
